# Image processing for AFB segmentation in bacilloscopies of pulmonary tuberculosis diagnosis

**DOI:** 10.1371/journal.pone.0218861

**Published:** 2019-07-15

**Authors:** Jorge Luis Díaz-Huerta, Adriana del Carmen Téllez-Anguiano, Miguelangel Fraga-Aguilar, José Antonio Gutiérrez-Gnecchi, Sergio Arellano-Calderón

**Affiliations:** 1 Departamento de Estudios de Posgrado en Ingeniería, Tecnológico Nacional de México / Instituto Tecnológico de Morelia, Morelia, Michoacán, México; 2 Laboratorio Estatal de Salud Pública, Morelia, Michoacán, México; University of California Berkeley, UNITED STATES

## Abstract

Image segmentation applied to medical image analysis is still a critical and important task. Although there exist several segmentation algorithms that have been widely studied in literature, these are subject to segmentation problems such as over- and under-segmentation as well as non-closed edges. In this paper, a simple method that combines well-known segmentation algorithms is presented. This method is applied to detect acid-fast bacilli (AFB) in bacilloscopies used to diagnose pulmonary tuberculosis (TB). This diagnosis can be performed through different tests, and the most used worldwide is smear microscopy because of its low cost and effectiveness. This diagnosis technique is based on the analysis and counting of the bacilli in the bacilloscopy observed under an optical microscope. The proposed method is used to segment the bacilli in digital images from bacilloscopies processed using Ziehl-Neelsen (ZN) staining. The proposed method is fast, has a low computational cost and good efficiency compared to other methods. The bacilli image segmentation is performed by image processing and analysis techniques, probability concepts and classifiers. In this work, a Bayesian classifier based on a Gaussian mixture model (GMM) is used. The segmentations' results are validated by using the Jaccard index, which indicates the efficiency of the classifier.

## Introduction

As indicated by Balandrano et al. in [1] the tuberculosis is considered one of the most important reemerging diseases and a public health problem worldwide, aggravated by the HIV epidemic and the drug resistance to tuberculosis. The World Health Organization (WHO) [2] estimates that one fourth of the world's population has latent tuberculosis.

Tuberculosis is a chronic, infectious and curable disease, which is caused in 95% of the cases by the bacillus *Mycobacterium tuberculosis* (*M*. *tuberculosis*) or Koch's bacillus. It is a rod-shaped aerobic bacillus measuring 2 to 6 μm long by 0.3 to 0.6 μm wide. According to Balandrano et al. [1] the main characteristic of the *M*. *tuberculosis* is being an acid-fast bacillus.

Balandrano et al. [1] establish that pulmonary tuberculosis is the most common type of the disease, comprising up to 80% of the cases. The transmission of the bacillus is almost exclusively airborne from person to person.

Tuberculosis diagnosis is the principle to solve this public health problem. Since 2000 more than 43 million lives have been saved thanks to the effective diagnosis and treatment of tuberculosis according to the WHO [3].

In 2017, TB caused an estimated 1.3 million deaths among HIV-negative people, and there were an additional 300 000 deaths from TB among HIV-positive people. Globally, the best estimate is that 10 million people developed TB in 2017: 5.8 million men, 3.2 million women and 1 million children. About 1.7 billion people, 23% of the world’s population, are estimated as having a latent TB infection and are thus at risk of developing active TB during their lifetime [2].

Different tests exist to diagnose pulmonary tuberculosis; however, many underdeveloped countries depend on sputum smear microscopy to diagnose tuberculosis since this test is fast, inexpensive, and ensures reproducible results with proper training.

By using an adequate staining technique, it is possible to identify the tuberculosis bacillus in the patient's sample; the Organización Panamericana de la Salud [4] mentions that two staining technique exist; auramine staining and Ziehl-Neelsen (ZN) staining. This paper focuses on the ZN staining technique because it is the most used worldwide and does not require special equipment; furthermore, it is recommended by the WHO and by the International Union against Tuberculosis and Lung Disease (IUATLD).

**Fig 1:**
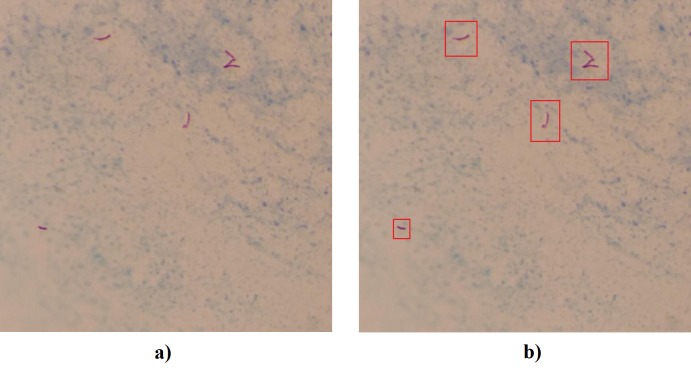
a) Typical sputum smear image; b) Bacilli highlighted in a red box.

In the ZN staining process, bacilli are dyed purple, while the background and other artifacts present in the bacilloscopy are stained blue. Fig 1A) shows the image of a typical sputum smear, in Fig 1B) the bacilli are highlighted by a red box to facilitate their identification.

Once the staining is done, the bacilloscopy is observed under an optical microscope, identifying and counting the bacilli. The degree of infection is determined according to the bacilli amount.

Processing the sputum smear should take into account diverse factors that influence the quality of the smear microscopy and therefore the diagnosis of the disease: sampling, staining technique, amount of dyes, and exposure time, among others. To obtain reliable laboratory results, both a correct technique and a good sample are required.

A specialized microscopist requires from 15 to 30 minutes to analyze a bacilloscopy depending on its characteristics. The large amount of smears analyzed by the microscopist in a single day make the analysis a tedious task, generating muscular and visual fatigue and, consequently, errors in the diagnosis.

Therefore, a system based on processing and analyzing smear images, which is capable of identifying AFB to help the pulmonary tuberculosis diagnosis by segmentation, may become an important tool in the health area because it can reduce the time required for analyzing the smear as well as reduce diagnosis errors, allowing the microscopists to increase the reliability and the number of daily performed diagnostics.

One of the main problems related to the automated bacilli detection is the digital image color and contrast variations, which affect the detection process’ sensitivity.

Sarkar et al. [5] present a ZN smear microscopy images’ database in order to facilitate the development of automated microscopy methods. Costa et al. [6] present a different sputum smear microscopy image database for automatically detecting bacilli in conventional microscopy.

There are several works reported in the literature to automatically detect bacilli from ZN smear microscopy images. Rulaningtyas et al. [7] present a color segmentation of sputum images using a Self-Organizing Map (SOM), which is compared to a k-means clustering method reported by Rico et al. [8] and Raof et al. [9]. The authors obtained 97.90% accuracy by using the SOM technique, whilst the k-means algorithm has 93.25% accuracy.

Shah et al. [10] performed a watershed segmentation method to detect bacilli images obtained from a camera-enabled smartphone microscope is presented. The authors report 93.3% sensitivity and 87% specificity.

Panicker et al. [11] present a method for detecting tuberculosis bacilli by image binarization and convolutional neural network segmentation. The method achieves 78.4% precision, 97.13% recall and 86.76% F-score in detecting TB.

An adaptive filtering is used to segment tuberculosis bacteria by Ayma et al [12]. The authors use Least Mean Squares and Reduced Rank with Eigen decomposition algorithms obtaining 70.92% and 70.89% correlation, respectively, and 93.52% and 93.56% recall, improving the 55.50% correlation and 49.25% recall obtained by using classical segmentation methods.

Sadaphal et al. [13] performed the segmentation using the color gradient applying a Bayesian classifier to determine the probability that a pixel corresponds to a bacillus. Dilation is applied to eliminate undesired artifacts, then an analysis of shape and size is performed to extract the characteristics of eccentricity and radius length for calculating the mean size and standard deviation. They use a classification tree to classify objects as bacilli, possible bacilli and false.

Payasi et al. [14] present a method for counting and detecting tuberculosis bacilli, converting from the RGB to the HIS color space, using the H channel information and applying a threshold of 175, as well as an area-based filter to eliminate artifacts. The authors use the bacilli width and length characteristics to label artifacts in the image as bacilli. Furthermore, the authors report an accuracy of up to 90%.

Mithra et al. [15] and Panicker et al. [11] present reviews of different image processing techniques applied to detecting tuberculosis bacilli from microscopic sputum smear images.

The revised works have results between 85% and 97.9% effectiveness; nevertheless, they have a high computational cost as well as a high processing time. In addition, the analyzed techniques still have important segmentation problems, independently of the application, as reported by Su et al. [16].

The main objective of this article is the implementation of a simple methodology that utilizes a Bayesian classifier based on a Gaussian mixture model (GMM) to segment tuberculosis bacilli, while obtaining a high detection effectiveness, as well as decreasing the processing time and the computational cost.

## Bacilli image segmentation

Segmentation is considered as the partition of an image in a set of non-overlapping regions, homogeneous with respect to a criterion whose union covers the whole image as indicated by Morales et al. in [17]. There are different methods to segment digital images.

**Fig 2:**
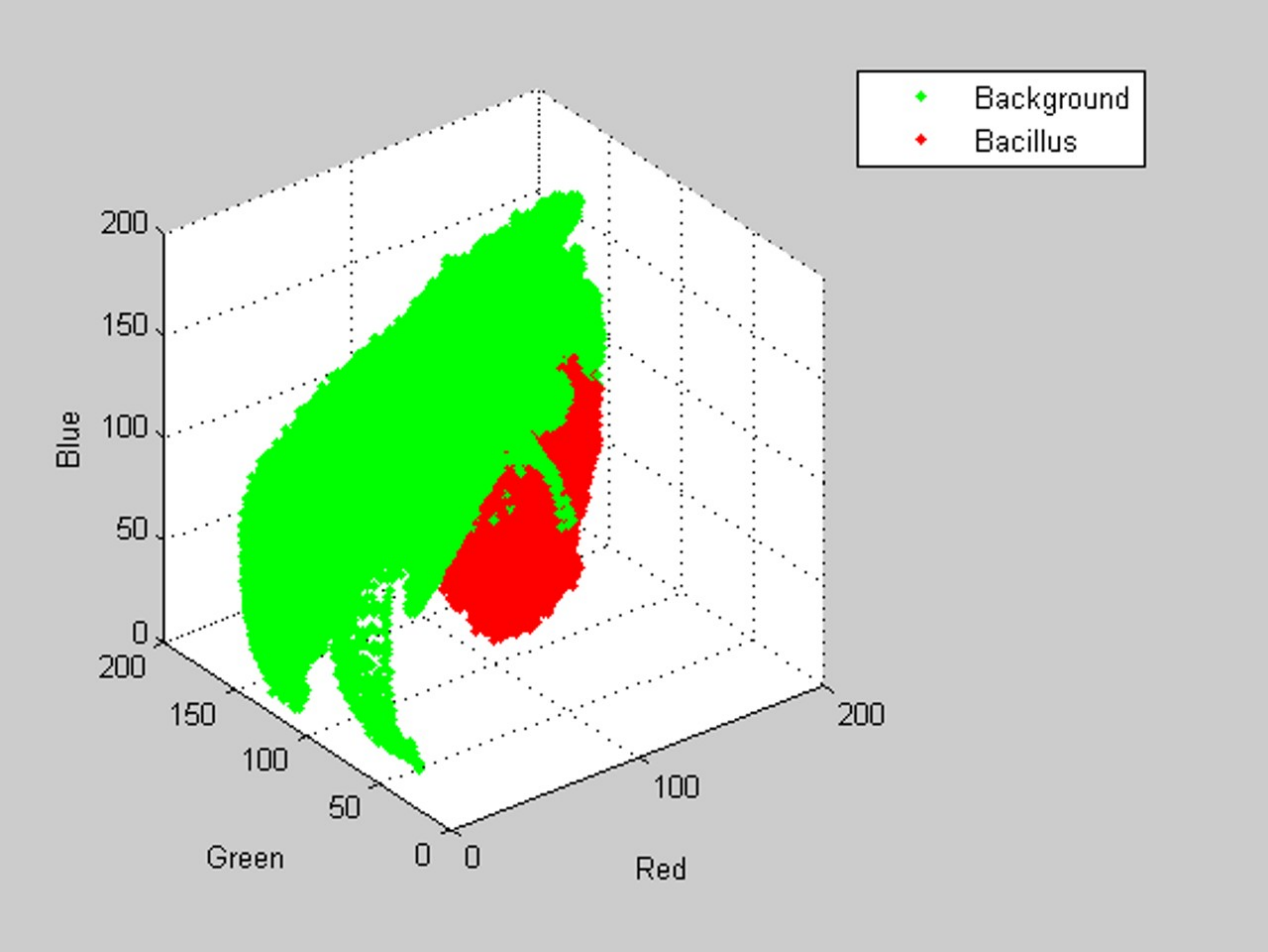
RGB data dispersion chart.

In this article two classes are considered, the bacillus class that includes all the data of the RGB components of the bacilli pixels and the background class that includes the data of the RGB components of the rest of the pixels. To perform the image segmentation, it is necessary to analyze the scatter plot of the data in the RGB plane presented in Fig 2.

By analyzing the scatter plot of the two classes, it is observed that there is no clear separation between the bacillus class and the background class. There is overlapping at different points, making it difficult to classify the data.

To solve this distribution problem, different combinations between color layers were tested in order to obtain a greater separation between classes. In addition, different segmentation methods were applied, such as logistic regression thresholds; however, because of the small separation between data classes, these tests were not successful.

### The Bayes classifier

The classification technique proposed in this paper is based on the Bayes theorem, which can be expressed as Eq (1):

$$p(C_{i}|x)=\frac{p(x|C_{i}).p(C_{i})}{p(x)}$$ (1)

where *p*(*C*_*i*_|*x*) is the likelihood that a pattern *x* belongs to the class *C*_*i*_, *p*(*x*|*C*_*i*_) is the likelihood that given the class *C*_*i*_ the value of the random variable is precisely *x*, *p*(*C*_*i*_) is the *a priori* likelihood that an element of the class *C*_*i*_ is manifested and *p*(*x*) is the *a priori* likelihood that a pattern *x* according to Morales et al. [17].

### The K-means algorithm

K-means is a clustering algorithm, which identifies groups or clusters of data points in a multidimensional space. The input for the k-means algorithm is a set of *N* data points *{x*_*n*_*…x*_*N*_*}*, each a random D-dimensional vector, and the number of clusters *K* the data set has to be divided into. The data points assigned to each cluster must have a smaller distance between them than with the members of other clusters. The *K* centers of each cluster are represented by the *D* dimensional vectors *μ*_*k*_ *= 1*,*…*, *K*.

In order to describe the assignment of a data point *X*_*n*_ to the *k* cluster, the set of binary indicator variables *r*_*nk*_*∈{0*,*1}* is used. If the data point *X*_*n*_ is assigned to the *kth* cluster, *r*_*nk*_ *= 1* and *r*_*nk*_ *= 0* otherwise. The objective function or distortion measure given by Eq (2) is the sum of the squares of the distances from each data point to its assigned center. The objective of the k-means algorithm is to find the values of the variables *r*_*nk*_ and *μ*_*k*_ that minimize (as presented in Eq (2)).

$$p(C_{i}|x)=\frac{p(x|C_{i}).p(C_{i})}{p(x)}$$ (2)

The distortion measure is minimized through an iterative process with two steps, the first minimizing with respect to the set of variables *r*_*nk*_ and keeping *μ*_*k*_ fixed, as well as keeping fixed *r*_*nk*_ and minimizing with respect to *μ*_*k*_. In the first step, the value of the distance from each point *X*_*n*_ to each center *μ*_*k*_ is independent of the other distances since both points are fixed in this step. Therefore, the objective function *J* is minimized by setting *r*_*nk*_ *= 1* for the value of *k* that gives the minimum *‖x*_*n*_*-μ*_*k*_*‖*^*2*^ and *r*_*nk*_ *= 0* for all other values of *k* and repeating for every *n*.

In the second step, *J* is minimized with respect to the center of the *kth* cluster *μ*_*k*_ by setting the gradient of *J* to zero to find the value of *μ*_*k*_ that minimizes *J* considering *r*_*nk*_ and the centers of the other fixed clusters. The previous procedure gives the expression for *μ*_*k*_ presented in Eq (3), which corresponds to the mean of the data points assigned to the *kth* cluster as stated by Bishop in [18].

$$\mu_{k}=\frac{\sum_{n=1}^{N}r_{nk}x_{n}}{\sum_{n=1}^{N}r_{nk}}$$ (3)

These two steps are repeated until there are no changes on the data points assigned to each cluster or after a given number of iterations has been exceeded.

### The expectation maximization algorithm

The Expectation Maximization (EM) algorithm is used to estimate the density distribution of the data points set of the same class, modeled as a mixture of Gaussian distributions.

A mixture of Gaussians is a linear combination of *K* Gaussian distributions of the form given in Eq (4), where *π*_*k*_, *μ*_*k*_ and *Σ*_*k*_ are the mixture coefficient, the mean vector and the covariance matrix of the *kth* multivariate Gaussian component. In order to normalize the mixture, the coefficients have to fulfill the restriction given by Eq (5) [18].

$$p(x)=\sum_{k=1}^{K}\pi_{k}N(x|\mu_{k}\Sigma_{k})$$ (4)

$$\sum_{k=1}^{K}\pi_{k}=1$$ (5)

The classic approach for choosing the set of the distribution parameters, *π*_*k*_, *μ*_*k*_, *Σ*_*k*_, is to find the values that maximize the probability of the data set (the likelihood). The likelihood function for a mixture of Gaussians has several local maximums. These difficulties are avoided in the EM algorithm by an iterative maximization procedure with two steps, similar to the k-means algorithm.

At the beginning, the parameters have random initial values, and the log of the likelihood is calculated using Eq (6). The two steps of EM are then repeated iteratively.

$$\ln\left(p(X)\right)=\sum_{n=1}^{N}\ln\left\{\sum_{k=1}^{K}\pi_{kn}(x_{n}|\mu_{k}\Sigma_{k})\right\}$$ (6)

The first step is the E step on which the responsibilities of the *k* Gaussian component are calculated according to Eq (7).

$$\gamma_{nk}=\frac{\pi_{k}N(x_{n}|\mu_{k},\Sigma_{k})}{\sum_{j=1}^{K}\pi_{j}N(x_{n}|\mu_{j},\Sigma_{j})}$$ (7)

The responsibilities are a probabilistic equivalent of the set of variables *r*_*nk*_ and indicate the fraction of the probability of *x*_*n*_ explained by the *kth* component of the mixture. The second step is called the M step, where the parameters are reevaluated using the responsibilities as stated in Eq (8). The E and M steps are repeated until convergence with the parameters or the log likelihood or after a maximum number of iterations is exceeded [18].

$$\mu_{k}=\frac{\sum_{n=1}^{N}\gamma_{nk}x_{n}}{\sum_{n=1}^{N}\gamma_{nk}},\;\Sigma_{k=}\frac{\sum_{n=1}^{N}\gamma_{nk}{\left(x_{n}-\mu_{k})(x_{n}-\mu_{k}\right)}^{T}}{\sum_{n=1}^{N}\gamma_{nk}}$$

$$\pi_{k}=\frac{\sum_{n=1}^{N}\gamma_{nk}}{N}$$ (8)

The Bayes classifier with the Gaussian mixture performs the segmentation pixel by pixel: therefore, by properly classifying each of the pixels, the bacilli present in the segmented images are formed.

In order to measure the efficiency of the classifier, the classic Jaccard index is used, which is a measure of similarity between two sets: the data set of the output image of the classifier and the data set of the original image segmented manually.

The Jaccard index depends on three simple incidence counts: the number of data shared by two sets and the number of unique data in each set. The classic Jaccard index for incidence counts is then Eq (9) as established by Chao et al. in [19]:

$$I_{j}:\;\frac{c}{\left(a+b-c\right)}$$ (9)

where *a* is the number of data present in set *A*, *b* is the number of data present in set *B* and *c* is the number of data present in both sets, *A* and *B*. This index is designed to be equal to 1 in cases of complete similarity and equal to 0 if the sets are dissimilar and do not have data in common.

## Materials and methods

**Fig 3:**
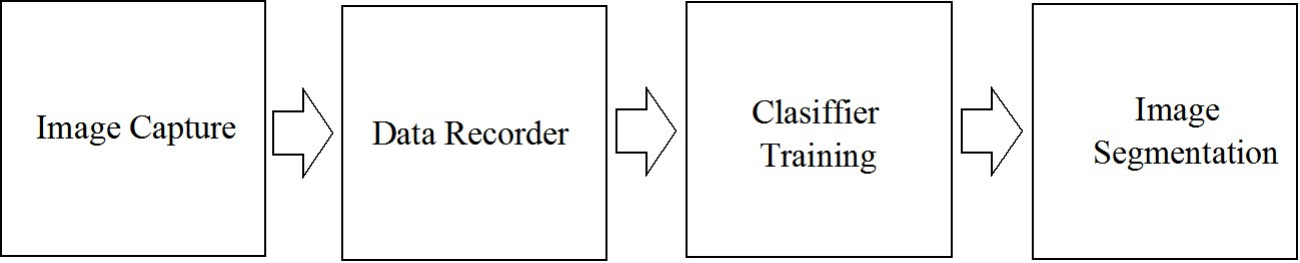
Bacilli detection process.

The process of performing the segmentation of bacilli in the images obtained from bacilloscopies of pulmonary tuberculosis is shown in Fig 3.

Digital images from smear bacilloscopies for diagnosing and controlling pulmonary tuberculosis were obtained from a Public Health Laboratory in Morelia, Michoacán, México, which provided the facilities, diagnostic and control bascilloscopies, an optical microscope, materials and reagents for the correct handling of the bacilloscopies, as well as trained personnel who provided support for the correct reading, bacilli identification and use of the laboratory equipment.

Choosing the appropriate digital camera is imperative because the bacilli data are extracted from the digital images: the better the images, the better the results.

There are special cameras for optical microscopes, however, their cost and little availability in public health centers make them a non-viable option for the implementation of this project. A feasible alternative is the use of the camera of a smartphone, widely used, to acquire the required digital images.

The selected device is a 13-MP smartphone’s camera, having the following characteristics: low cost, high resolution, high color quality and contrast between the background and the bacilli.

**Fig 4:**
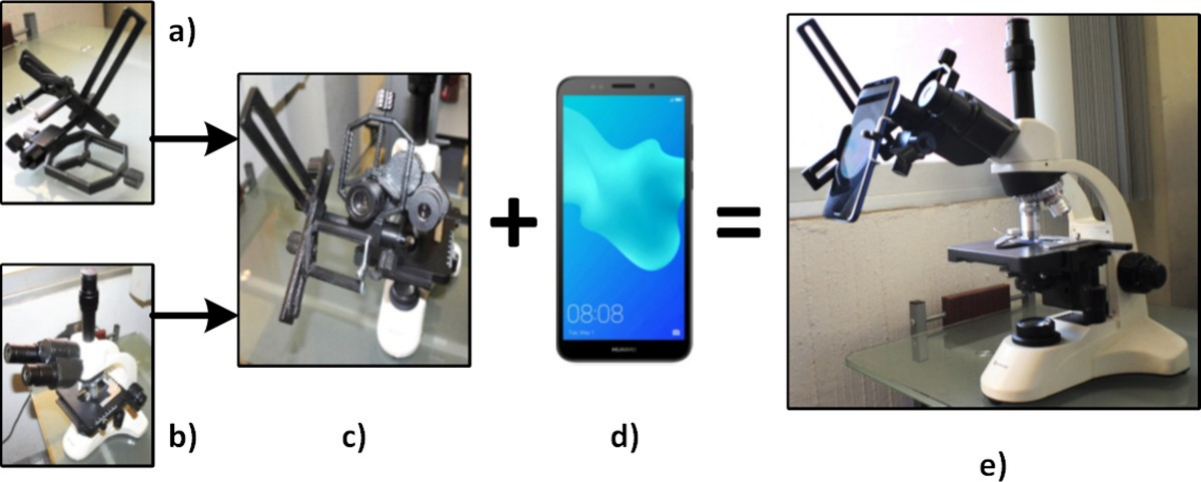
Smartphone mounting: a) Adapter, b) Optical microscope, c) Adapter-Microscope mounting, d) Smartphone, e) Image capture system.

In Fig 4 the smartphone mounting required to capture digital images is presented. The process of mounting requires an adapter (Fig 4A) mounted in the ocular of a standard optical microscope (Fig 4B) as shown in Fig 4C. Once the adapter is mounted, the selected smartphone (Fig 4D) is located into the adapter to complete the capture system shown in Fig 4E. The position adjustments are obtained by moving the corresponding screws of the adapter in order to capture the largest possible area of the visible field. To correctly focus the smear images, the macrometric and micrometric knobs of the microscope are used. However, as the adapter is manually adjusted to the microscope, this system has the limitation of lacking a fixed location provoking small variations in the size of the bacillus in the image.

In order to perform the segmentation algorithm, a personal computer was used. The computer characteristics are: Intel(R) Core (MT) i5-3230 CPU@2.60 GHz 2.60 GHz processor, RAM 8 GB, 64-bit operating system, and Windows 7 Ultimate, which are commonly used.

Although it is possible to perform the processing step with the smartphone, the personal computer is used because this research is part of a complete project that requires further mathematical processing. In addition, the microscopist (final user) needs to visualize the processed images in the largest size possible.

### Image capture and data record

Approximately 200 images were obtained from 30 bacilloscopies processed with the ZN staining method. Because the size of the bacilli is between 2 to 6 μm, a 100x microscope’s objective lenses were selected, which allowed to obtain a visual field of the smear of 180 μm in diameter.

**Fig 5:**
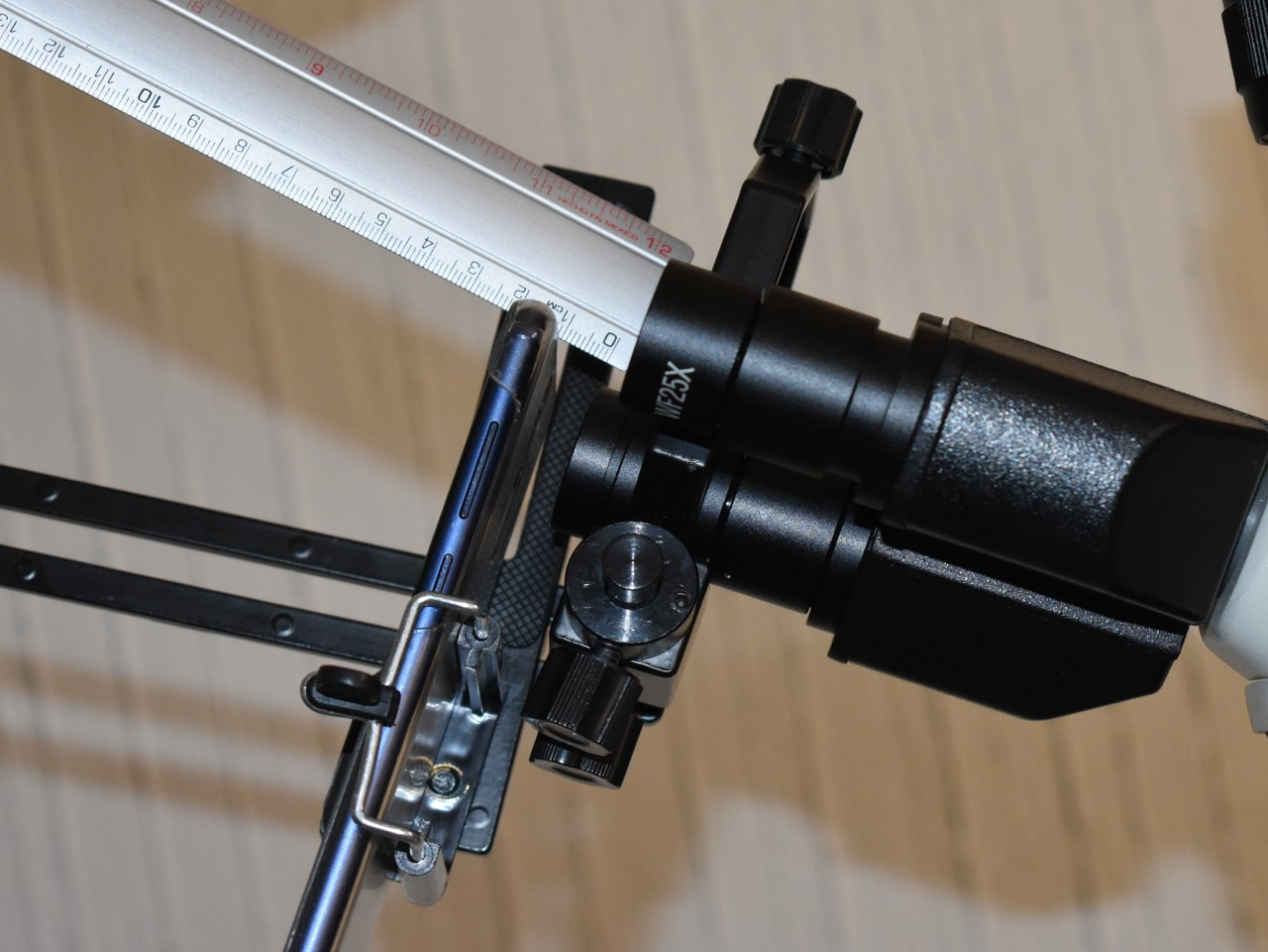
Distance between the microscope’s ocular and the smartphone lens.

In order to obtain similar sizes and resolutions in the images taken, the distance between the microscope ocular and the smartphone was measured and placed in *x = 1 cm*, *y = 0* and *z = 0*, according to fixed reference points in both devices. Fig 5 shows the selected distance by presenting a smear as a size reference object.

**Fig 6:**
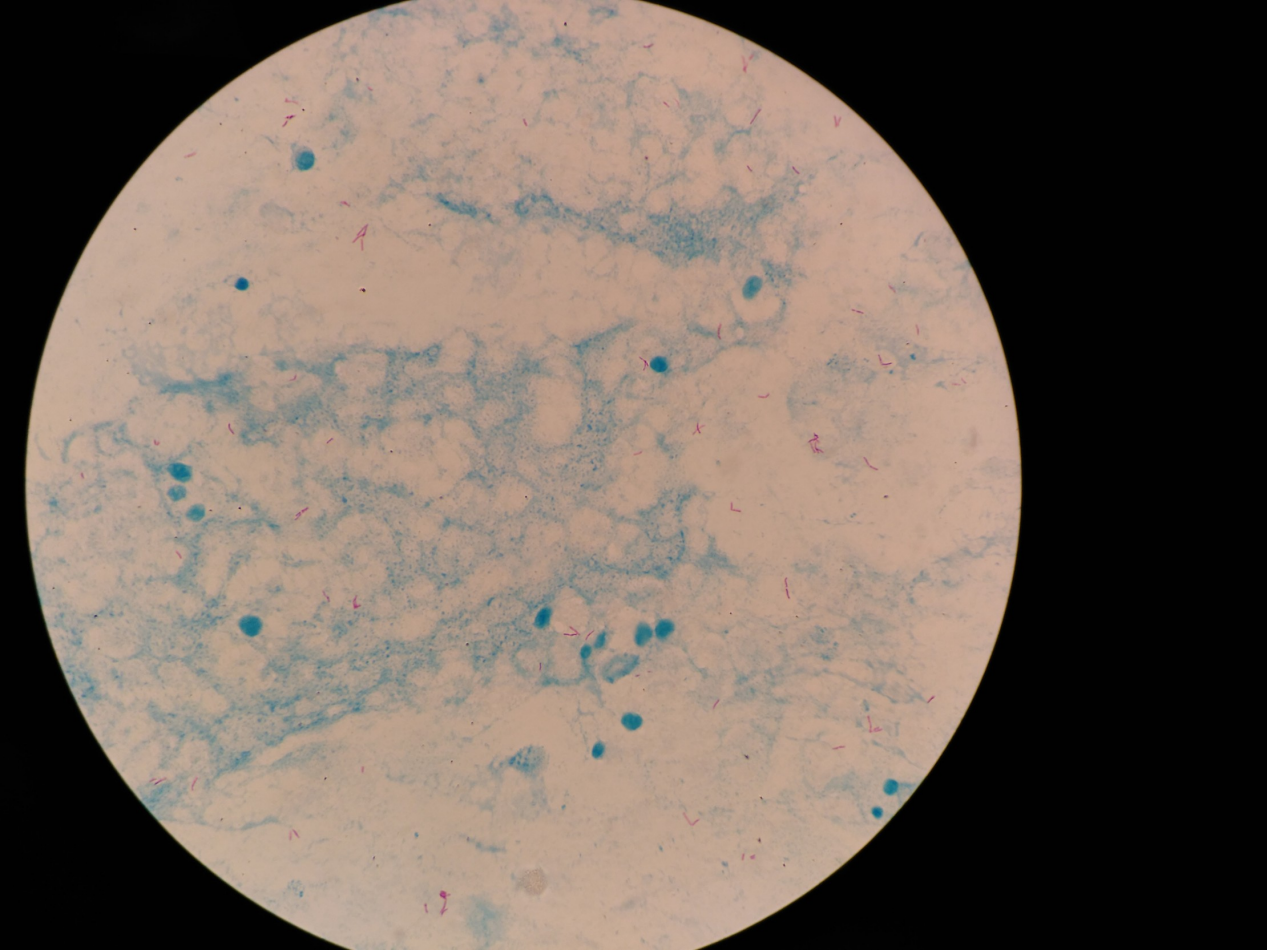
Original microscope image. 4160*3120 pixels.

Fig 6 shows an original image obtained by the smartphone’s camera located at the selected distance from the microscope’s ocular; this distance allows capturing almost the whole visual field. Although it is possible to obtain the entire visual field, a small part of it is sacrificed to favor the size of the section to analyze.

The size of the image obtained by the smartphone is 4160*3120 pixels; thus, by measuring the image, the diameter of the visual field corresponding to a 180 μm is equivalent to 3260 pixels.

**Fig 7:**
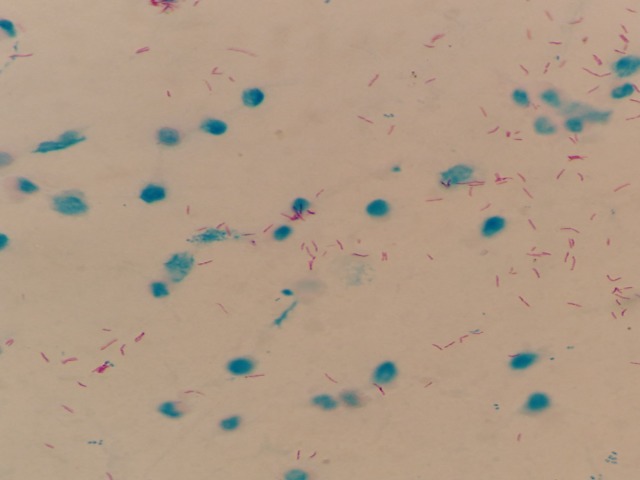
Cut microscope image. 2513*2073 pixels.

Because the entire image has black areas, which are undesirable for performing an adequate segmentation process, a rectangular area is cut out from the center of the image. The selected area measures 2513*2073 pixels, which is equivalent to 138.75* 114.46 μm of the visual field. The new image (Fig 7) is saved in order to start the segmentation process.

### Data record

The selected data of the images were obtained by using the GIMP 2.0 and the MATLAB programs. Pixels belonging to the bacilli class were selected manually using the GIMP program; later a program created in MATLAB was used to extract the RGB values of each pixel, and a database was created containing these values, as well as a label “1” indicating the bacillus class.

GIMP was then used to select background images with different coloration characteristics, as well as existing artifacts. The RGB values were obtained by using a MATLAB program; furthermore, the obtained data were stored in a database and assigned a label “0” for being identified as the background class.

### Classifier training and image segmentation

According to the analysis of the scatter plot, a Bayes classifier with Gaussian mixture was chosen. The *NetLab3_3* library was used to perform the training of Gaussian mixtures for both, the background and the bacillus class. The obtained data were stored in a new database.

The K-means algorithm is used as a first approximation method to provide the EM algorithm with better initial values for the Gaussian mixture distribution parameters than random values, at very little computational cost, thus reducing the number of iterations required by the EM algorithm to converge.

Once the training of the Bayes classifier with the Gaussian mixture is done, the database containing the previous results is loaded, and calculations of the posterior probability function of the bacillus class and the background class are performed by using a Gaussian mixture model, which assigns the analyzed pixel to the class with the highest probability. In other words, the Bayesian classifier calculates the probability that a pixel belongs to the bacillus class or the background class and assigns it to the class with the highest probability.

The number of centers is determined by performing different tests in order to find the combination that presents the best results. In this article, six Gaussians are determined for the bacillus class and four for the background class.

## Results and discussion

**Fig 8:**
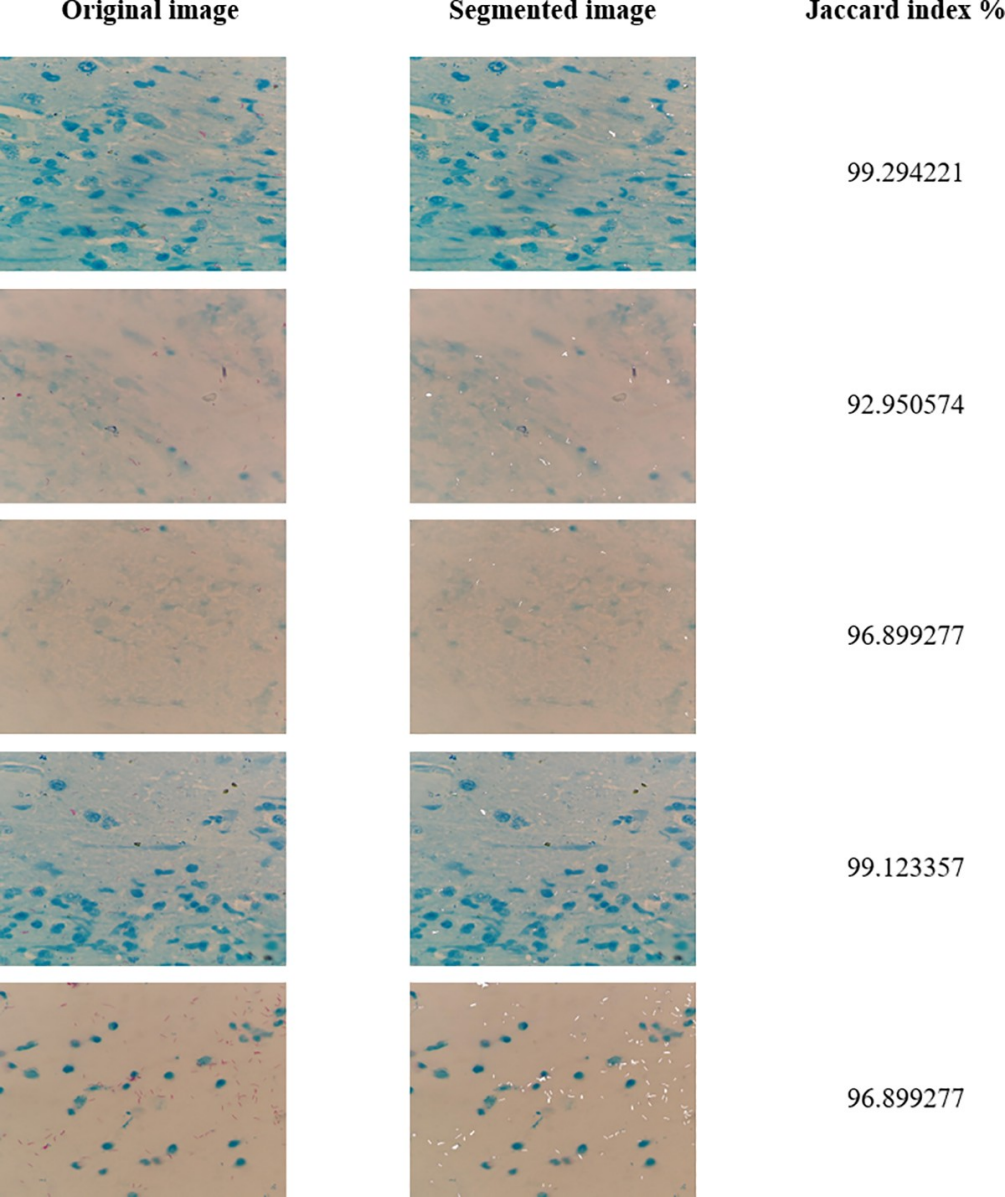
Segmentation results.

The obtained results are shown Fig 8. The original digital images obtained from the bacilloscopies are shown in the left column of Fig 8, while the segmented images are presented in the middle column. The column in the right show the calculated Jaccard indexes of the segmented images.

The segmentation accuracy is measured by the Jaccard index, column C, with a calculation that considers that the white pixels belong to the bacillus class, whereas the rest of the pixels are categorized as background class.

In this study, more than 200 images were analyzed and it was concluded that they could be clustered according to the most common hue and contrast characteristics in the following classes: adequate, little blue and / or purple, excess of blue and / or purple, bacilli stained in both, blue and purple colors. These different types have equal prevalence and it was observed that similar characteristics of the images have similar Jaccard indexes.

Only five representative images of the different types are reported. Each image was segmented in approximately 7 seconds using the described computer.

### Image resolution analysis

In order to guarantee an adequate bacilli detection, an image resolution analysis, related to the minimum pixel size in micrometers to this end, was performed.

The 2513*2073-pixel image is reduced to different standard resolutions, which modifies the size in pixels of the image, while the size in μm of the useful field remains constant. Table 1 shows the number of pixels per μm^2^ for each resolution.

**Table 1:** Images' sizes.

Image size (pixels)	Visual field size (μm)	Pixels per μm^2^
2513*2073	138.7*114.4	≈18*18
1920*1080	138.7*114.4	≈ 14*9
1280*960	138.7*114.4	≈ 9*8
800*600	138.7*114.4	≈6*5
640*480	138.7*114.4	≈4*4

In order to determine the minimum resolution required to obtain an adequate bacilli segmentation, the accuracy percentage of the bacilli pixels detected correctly is used. The accuracy percentage, *%P*, was determined by (10).

$$\%P=100-(\frac{msp-asp}{msp}*100)$$ (10)

where *msp* is the number of pixels corresponding to the bacilli class segmented manually (accuracy reference) and *asp* the number of pixels corresponding to the bacilli class segmented by the algorithm.

Table 2 shows the segmentation results corresponding to each resolution. According to the obtained results, the minimum resolution recommended for performing an accurate segmentation is 2513*2073 pixels.

**Table 2:** Segmentation results considering different image sizes.

Image size (pixels)	Accuracy percentage (a)
2513*2073	98.6602
1920*1080	70.5610
1280*960	59.3452
800*600	36.4960
640*480	22.4754

## Conclusion

The obtained results allow to conclude that the bacilli segmentation in the digital image by using the proposed methodology has up to 92% effectiveness, under different color and contrast image conditions. For normalized images, the method provides up to 98% effectiveness.

The bacilli detection can be performed based on these segmentation results, helping to identify the bacilli by shape and size.

The presented method is considered reliable and is an improvement on the results reported in the literature (85 to 97% effective), which do not consider variations in the color and contrast of the processed images.

The time used in the segmentation process is approximately 7 seconds for each original image of 2513 x 2073 pixels; this time includes the display of three figures obtained during the processing steps (approximately 1 second per figure), which makes a fast, reliable tool with a low computational cost for detecting bacilli.

The method is reproducible as long as the images have characteristics similar to those shown in this article because the coloration and contrast in the images obtained from the smear depend on several factors, such as the elaboration of the smears, reagents used, the dye process, and the camera used, among others.

In order to increase the robustness of the system, it is necessary to perform preprocessing tasks to eliminate such variability by standardizing the RGB components of the image.

In addition, it is necessary to consider the image resolution in order to obtain adequate segmentation results.
